# Growth strains cause vascular browning and cavities in ´Nicoter´ apples

**DOI:** 10.1371/journal.pone.0289013

**Published:** 2023-07-20

**Authors:** Eckhard Grimm, Merle Peters, Julian Kaltenbach, Chu Zhang, Moritz Knoche

**Affiliations:** Institut für Gartenbauliche Produktionssysteme, Abteilung Obstbau, Leibniz Universität Hannover, Hannover, Germany; Monash University Malaysia, MALAYSIA

## Abstract

‘Nicoter’ apples (*Malus* × *domestica* Borkh.) occasionally develop a disorder referred to as vascular browning. Symptomatic fruit are perceived as being of low quality. The objective was to identify the mechanistic basis of this disorder. The frequency of symptomatic ‘Nicoter’ apples differed between growing sites and increased with delayed harvest. Typical symptoms are tissue browning and cavities in the ray parenchyma of the calyx region, and occasionally also of the stem end. Cavity size is positively correlated with the extent of tissue browning. Cavities were oriented radially in the direction of the bisecting line between the radii connecting the calyx/pedicel axis to the sepal and petal bundles. Microscopy revealed cell wall fragments in the cavities indicating physical rupture of cell walls. Immunolabelling of cell wall epitopes offered no evidence for separation of cells along cell walls. The growth pattern in ‘Nicoter’ is similar to that in its parents ‘Gala’ and ‘Braeburn’. Allometric analyses revealed no differences in growth in fruit length among the three cultivars. However, the allometric analyses of growth in diameter revealed a marked increase in the distance between the surface of the calyx cavity and the vascular bundle in ‘Nicoter’, that was absent in ‘Braeburn’ and ‘Gala’. This increase displaced the petal bundles in the ray parenchyma outwards and subjected the tissue between the petal and sepal bundles to tangential strain. Rupture of cells results in tissue browning and cavity formation. A timely harvest is a practicable countermeasure for decreasing the incidence of vascular browning.

## Introduction

‘Nicoter’ apples are marketed under the label Kanzi®. Kanzi®/‘Nicoter’ and have become an important, upmarket apple cultivar in both the Northern and Southern Hemispheres [1, 2]. Key characteristics are said to be an attractive appearance, and excellent firmness and taste [3]. However, ‘Nicoter’ apples occasionally suffer from a disorder commonly referred to as ‘vascular browning’ [2]. In contrast flesh and core browning [4–9], the typical symptoms of vascular browning are tissue browning and the formation of cavities close to the calyx and the stem ends of the fruit [2]. Vascular browning is perceived as an important quality defect of ‘Nicoter’ [2].

Symptoms of vascular browning are not detectable by the naked eye from the outside. Also, the small size of the affected tissue and the immediate vicinity to calyx and pedicel make it difficult to detect fruit with vascular browning during grading in the packhouse. Hence, symptomatic fruit cannot be excluded reliably from the market. Growers observations suggest that cavities develop preharvest and are plainly visible from harvest onwards when fruit are sectioned in the calyx region. The frequency of occurrence of vascular browning of ‘Nicoter’ varies from year to year, from region to region and from orchard to orchard. Anecdotal evidence relates the frequency of occurrence of vascular browning to seasonal effects. The incidence is said to increase with delayed harvest date. However, systematic scientific studies are lacking. To develop suitable counter strategies for vascular browning requires a better understanding of its incidence and its mechanism.

The objective of this study was to describe the anatomical basis of the vascular browning disorder of ‘Nicoter’ apples and to identify its mechanistic basis. We focused on the following aspects:

1. The morphological and histological characteristics of symptomatic and asymptomatic ‘Nicoter’ apples in comparison to its parents ‘Braeburn’ and ‘Gala’,
2. The cell wall properties of the cortical tissues of ‘Nicoter’, ‘Braeburn’ and ‘Gala’ apples, and
3. The patterns of growth and development of ‘Nicoter’, ‘Braeburn’ and ‘Gala’ apples.

## Material and methods

### Plant material

Batches of symptomatic mature ‘Nicoter’ apple were obtained from commercial fruit growers at Neuenfelde (53.5N, 9.78E) in northern Germany and Überlingen (47.7N, 9.1E) in southern Germany. Developing ‘Nicoter’, ‘Braeburn’ and ‘Gala’ apples (*Malus ×domestica* Borkh.) were sampled randomly in a commercial orchard at Ohndorf, Germany (52.3N, 9.3E) at 2- to 4-week intervals, beginning 14 days after full bloom (DAFB) until commercial maturity (optimum harvest date). The effect of a 6-week delay in harvest date was established using fruit from the Ohndorf site. Fruit were sampled at commercial maturity (optimum harvest date) and six weeks thereafter (delayed harvest).

All trees were grafted on M9 rootstocks and cultivated according to current regulations for integrated fruit production. Fruit were held in cold storage at 1–3°C and a relative humidity of 80−90% for a maximum of one month before analysis. The only exception was the ‘Gala’ used for immunolabelling that was held at 1.7°C, 93% RH, 4.8% O_2_ and 0.9% CO_2_ for a maximum of 40 days.

### Promalin application

The effect of Promalin treatment (Valent BioSciences, Libertyville, Ill 60048, USA) on the incidence vascular browning was established at Ohndorf. These fruit were harvested from four trees that were sprayed with Promalin (0.8 g l^-1^) at full bloom (0.75 l per tree) and at 9 (1.25 l per tree) and 30 DAFB (2.5 l per tree). Fruit from unsprayed trees served as control.

### Macroscopic analysis

The frequencies of vascular browning and cavity formation were established using mature ‘Nicoter’ apples from Neuenfelde, Überlingen and Ohndorf, from fruit without and with the delayed harvest (Ohndorf) and from Promalin treated fruit (Ohndorf).

Cross-sections were prepared in the calyx and the pedicel-end regions and the numbers of symptomatic fruit were counted.

The numbers of symptomatic fruit and of symptomatic sectors within a fruit were recorded. A sector is defined as a fruit segment excised between two adjacent vascular bundles (i.e., one tenth of 360°, so equivalent to a 36° segment).

The number of cavities per fruit, and the cavity volumes, positions and orientations were quantified in ‘Nicoter’ fruit from Überlingen. Cross-sections were prepared in the calyx region and the number of cavities counted. Casts of the cavities were prepared by filling with silicone rubber (DOWSIL™SE 9186 L, Dow, Midland, USA). After curing, the silicone rubber was removed and weighed, and the volume of the cast calculated assuming a density of the silicone rubber of 1.02 kg dm⁻^3^. The volume of the tissue affected by browning was quantified by carefully carving out and removing the brown tissue surrounding the cavity. Thereafter, a cast of the newly formed enlarged cavity was prepared using the procedure described above. The volume of brown tissue was taken as equal to the difference in the volumes of the original cavity and of the enlarged cavity.

To assess a potential role of the seeds in vascular browning, the number of seeds per locule was established for the locules underlying cavities, for locules neighboring cavities and for locules on the opposite side to the cavity.

### Microscopic analysis

Longitudinal sections parallel to the calyx/pedicel axis and cross-sections perpendicular to the calyx/pedicel axis were prepared in the calyx region of ‘Nicoter’ from Überlingen and Ohndorf using a razor blade. Sections were stained with 0.05% toluidine blue or 0.01% methylene blue and embedded in silicone oil. Sections were viewed in incident bright light using a microscope (MZ10F; Leica Mikrosysteme, Wetzlar, Germany) equipped with a digital camera (DP 73; Olympus Europa, Hamburg, Germany). Calibrated images were taken and analyzed using image analysis software (cellSens Dimensions; Olympus Europa, Hamburg, Germany). The positions and orientations of the cavities relative to the vascular bundles were quantified.

### Immunolabelling

Cell wall epitopes exposed on the cut surface (cross-sections) of symptomatic and asymptomatic ‘Nicoter’ (all fruit from Ohndorf) and those exposed on artificial longitudinal fracture surfaces of ‘Nicoter’, ‘Braeburn’ and ‘Gala’ were studied by immunolabelling using monoclonal antibodies (mAbs). The cross-sections were prepared at about the point of inflection of the vascular bundles in the calyx region from a minimum of three fruit per cultivar. They comprised the cut surface with pith, vascular bundles, ray parenchyma and cortex. Artificial fracture surfaces were produced by manually fracturing tissue sections of the outer cortex in a radial direction. Tissue blocks (5 x 5 x 2 mm) were excised that contained the fracture surface for immunolabelling. The tissue blocks were stored at 4°C in 70% ethanol for up to 9 days. For immunolabelling, the protocols of [10–12] were followed with minor modifications. The tissue blocks were washed twice in isotonic phosphate-buffered saline (PBS) solution for 5 min each. Non-specific protein binding sites were blocked using 3% nonfat dry milk powder prepared in PBS. The blocking solution was then removed and the tissue washed three-times for 5 min each using PBS. The primary monoclonal antibodies (PlantProbes, Leeds, UK) were used at a dilution of 1:10 in 10 mM PBS: LM7 (anti-homogalacturonan) [13], LM8 (anti-xylogalacturonan) [14], LM19 (anti-homogalacturonan) [15], LM20 (anti-homogalacturonan) [15] and 2F4 for dimeric associations of Ca with homogalacturonan [16]. Those reacting with hemicelluloses were: LM11 (anti-xylan/arabinoxylan) [17], LM21 (anti-mannan) [18] and LM25 (anti-xyloglucan) [19]. Only the mAB 2F4 was buffered in 20 mM Tris(hydroxymethyl)methylamin buffer (TRIS-HCl) adjusted to pH 8.2. All primary mAbs were anti-rat, only 2F4 was anti-mouse. The mAbs were allowed to bind to the epitopes of the tissue for 1 h followed by three washing steps with PBS. The bound antibodies in the tissue were then reacted in the dark with a secondary antibody carrying the fluorescence marker Alexa Fluor 488 (Lifetechnologies, Eugene, Oregon, USA) either bound to an anti-rat antibody (LM series) or to an anti-mouse antibody (2F4). The secondary antibody was prepared in PBS at a 100-fold dilution. Unbound antibodies were removed by washing twice in PBS. To identify cellulose in the cell walls, the tissue was subsequently stained with calcofluor white (fluorescent brightener 28; Sigma-Aldrich Chemie, Munich, Germany) for 5 min, followed by two washing steps. The tissue was then transferred to a microscope slide on the stage of a microscope, treated with an antifading solution (Citifluor AF3; Science services, Munich, Germany) made up in PBS, covered with a cover slip and then viewed. Calibrated micrographs were taken at a magnification of 2× using a fluorescence binocular microscope (MZ10F equipped with a GFP-plus filter: 480–440 nm excitation, ≥510 nm emission wave length and a UV filter: 360−440 mm excitation, ≥420 Emission wave length; Leica, Wetzlar, Germany) and a digital camera (DP73; Olympus, Hamburg, Germany).

### Growth analysis

Allometric relationships between various dimensional measures of fruit growth were analyzed in developing ‘Nicoter’, ‘Braeburn’ and ‘Gala’ apples from Ohndorf. Fruit were weighed, cut longitudinally along the calyx pedicel axis and the sections photographed on a photostand (camera EOS 550D; Canon, Tokio, Japan; photostand: RT1, Kaiser Fototechnik, Buchen, Germany). The calibrated images were analyzed using image analysis software (cellSens Dimensions, Olympus Europa, Hamburg, Germany). The following dimensions were quantified: maximum fruit diameter (D), maximum fruit length height (L); distance between calyx lob and base of sepal (La), distance between base of sepal and point of inflection (ip) of vascular bundle (Lb), distance between point of inflection of vascular bundle and distal end of locule (Lc), distance between calyx-stem axis and surface of calyx cavity (Ld), distance between surface of calyx cavity and point of inflection of vascular bundle (ip) (Le), distance between point of inflection of vascular bundles and fruit surface (Lf) (see Figs 8 and 9).

### Tensile tests

Dumbbell shaped specimens were prepared from the outer cortex and clamped in a custom-made holder [20]. The outer cortex was used because it was technically impossible to produce fracture surfaces in the ray parenchyma in the vicinity of the vascular bundles where most cavities occurred. Clamping distance was 10 mm, the thickness of the specimen was 3 mm, the waist width 5 mm. The holder was constructed such that unintentional stress and damage to the specimen during handling and clamping was minimized. The specimen was mounted between the clamps of a material testing machine (Z 0.5; Zwick/Roell, Ulm, Germany; 10 N force transducer, Type: KAP-Z; Zwick/Roell). A uniaxial tensile force was applied at a constant strain rate of 3 mm min^-1^ until failure. The applied force (N) and distance (mm) of the movement of the crosshead were recorded. The stress (N mm^-2^) applied was calculated by dividing the force by the cross-sectional area of the waist of the specimen (15 mm^2^) [21]. The extension of the specimen was calculated as the relative elongation by dividing the actual length (mm) by the original length (10 mm). The modulus of elasticity (*E* in N mm^-2^) was calculated as the maximum slope of the stress (N mm^-2^) vs. fractional strain (%/100) relationship [21]. The number of individual fruit replicates was 12 with two specimens tested per fruit.

### Data analysis

Data are presented as means ± standard errors of the mean. Data were analyzed by regression analysis using the statistical software package SAS (version 9.4; SAS Institute, NC, USA). The Figures were prepared using the software package Sigmaplot 12.5 (Systat Software GmbH, Erkrath, Deutschland).

## Results

### Macroscopic and microscopic analyses

**Fig 1:**
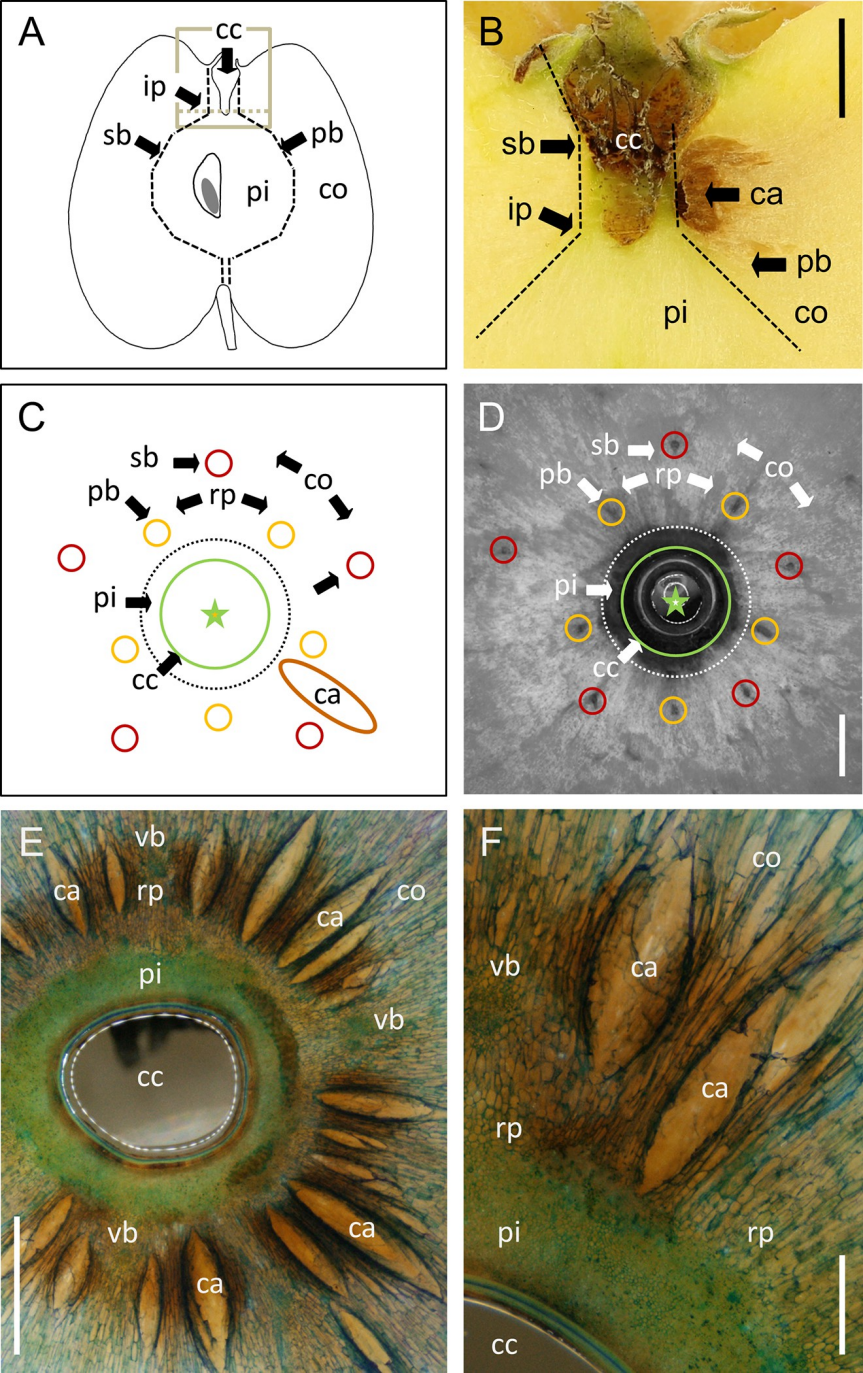
Sketches (AC) and micrographs of longitudinal sections (B) and cross-sections (D-F) through mature ‘Nicoter’ apples. (A) Sketch of longitudinal section of the whole fruit. Grey box indicates detailed view of calyx region shown in micrograph in (B). Abbreviations are: cc, calyx cavity; co, cortex; pi, pith; sb, sepal bundle; pb, petal bundle; ip, point of inflection of vascular bundles; ca, cavity with surrounding browning of cortex. (C) Sketch of cross-section of fruit in calyx region and corresponding micrograph (D). Five sepal bundles (sb, red) and five petal bundles (pb, yellow) are arranged in two circles of differing radii with the sepal bundles being the outermost. Pith (pi) with small cells is separated from elongated cells (dotted line) of the ray parenchyma (rp). ca, cavity in symptomatic fruit; cc, calyx cavity as indicated by green circle. The asterisk in the center indicates the fruit axis. (EF) Cross-sections overview (E) and detail (F) of symptomatic ‘Nicoter’ apple having numerous cavities (ca) surrounded by brown tissue. cc, calyx cavity; co, cortex; pi, pith; rp, ray parenchyma; vb, vascular bundle. Scale bars in (A-C) 5 mm, in (D, E) 2 mm and in (F) 0.5 mm. Images were obtained from a minimum of ten symptomatic apples.

‘Nicoter’ apples affected by the vascular browning exhibited tissue browning with or without cavity formation (for view of whole fruit see S1 Fig; Fig 1B, 1E and 1F).

The frequency of symptomatic ‘Nicoter’ apples depended on the growing site and was highest in Neuenfelde and lowest in Ohndorf (Table 1). Symptoms occurred more frequently in the calyx end, as compared to the stem end. The application of promalin had no effect on the incidence of tissue browning or of cavity formation.

**Table 1:** Frequency of ‘Nicoter’ apples affected by vascular browning at three different sites without and with application of promalin.

Site	Number of fruit	Frequency of symptomatic fruit (%)			
		Total	Calyx	Stem end	Calyx + stem end
Neuenfelde	170	82	73	51	42
Überlingen	50	60	56	14	10
Ohndorf (control)	50	6	2	4	0
Ohndorf (Promalin)	50	4	2	2	0

The frequency (%) was calculated by dividing the number of symptomatic fruit by the total number of fruit inspected.

Delaying the harvest of ‘Nicoter’ apples by six weeks beyond the optimum harvest date markedly increased the frequency of symptomatic fruit both, on a whole-fruit basis, but also on a segment basis (Table 2).

**Table 2:** Effect of harvest date on the frequency of vascular browning in ‘Nicoter’ apples. Fruit were harvested either at the optimum harvest date (‘optimum’) or six weeks later (‘Delayed by 6 weeks’). Vascular browning was assessed both on a whole-fruit basis and on an individual-segment basis where one segment corresponds to a tissue segment bounded by two adjacent vascular bundles.

Harvest date	Number of fruit	Frequency of symptomatic fruit (%)	Number of segments	Frequency of symptomatic segments (%)
Optimum	20	20	200	5
Delayed by 6 weeks	20	90	200	54

The calyx end of ‘Nicoter’ apples comprised (from inside to outside) the calyx cavity, the pith (pi), vascular bundles connecting to the sepals (sb) and petals (pb), ray parenchyma (rp), and the cortex (co) (Fig 1A and 1C). The pith consisted of small isodiametric parenchyma cells or small elongated ones that were stretched in a direction parallel to the calyx-stem axis. Generally, the pith was free of any symptoms (Fig 1D and 1F). The cross-section in the calyx region revealed ten vascular bundles (vb) surrounding the pith. The ten vascular bundles were embedded in ray parenchyma and connected alternately to the sepals (sb) or to the petals (pb) (Fig 1C and 1D). The parenchyma outside the vascular zone formed the cortex (co) (Fig 1C and 1D). Both ray and cortical cells were radially elongated with a radially-increasing ratio of length to width (the aspect ratio) (Fig 1D and 1F). In symptomatic ‘Nicoter’, tissue browning and cavities were located close to the inflection point (ip) of the vascular bundles (Fig 1B). Tissue browning and cavities occurred in the ray parenchyma and the cortex (Fig 1E and 1F), but rarely in the pith. Cavities were always associated with browning of the surrounding tissue. The cavities were mostly radially elongated and diagonally orientated to the calyx-stem axis. They had a spindle-type shape, their size was variable (Fig 1E and 1F). In severely affected fruit, numerous cavities appeared as a star with the calyx cavity located at its center (Fig 1F).

**Fig 2:**
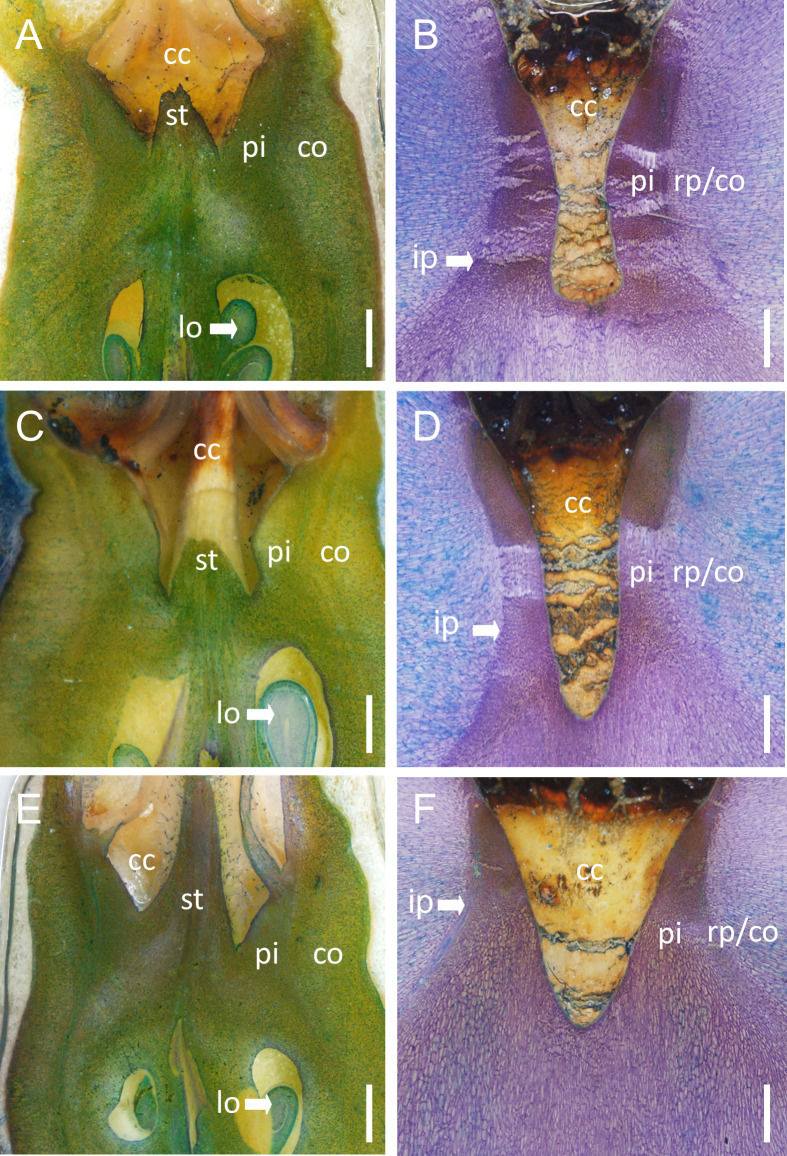
Light micrographs of longitudinal sections of ‘Nicoter’ (AB), ‘Gala’ (CD), ‘Braeburn’ (EF) apples in the calyx region during early (14 to 17 DAFB, days after full bloom; ACE) and late fruit development (122 to 153 DAFB; BDF). Sections were stained with toluidine blue and subsequently viewed in incident light. cc, calyx cavity; st, remains of the style; lo, locule with ovules; pi, pith; rp, ray parenchyma; co, cortex; ip, point of inflection of vascular bundles. Scale bars in (A-F) 1.0 mm. A minimum of eight fruit per cultivar was inspected.

Microscopy of the calyx region (Fig 2) revealed significant cell elongation in the tissues surrounding the calyx cavity. In early stages of fruit development, cells were isodiametric (Fig 2A, 2C and 2E), in later stages (Fig 2B, 2D and 2F) the major directions of cell extension were radially (latitudinally perpendicular to the central axis) relative to the central axis of the fruit in the pith, but longitudinally parallel to the central axis towards the calyx cavity in the ray parenchyma and the cortex. In these regards, there were few differences between ‘Nicoter’, ‘Gala’ or ‘Braeburn’.

**Fig 3:**
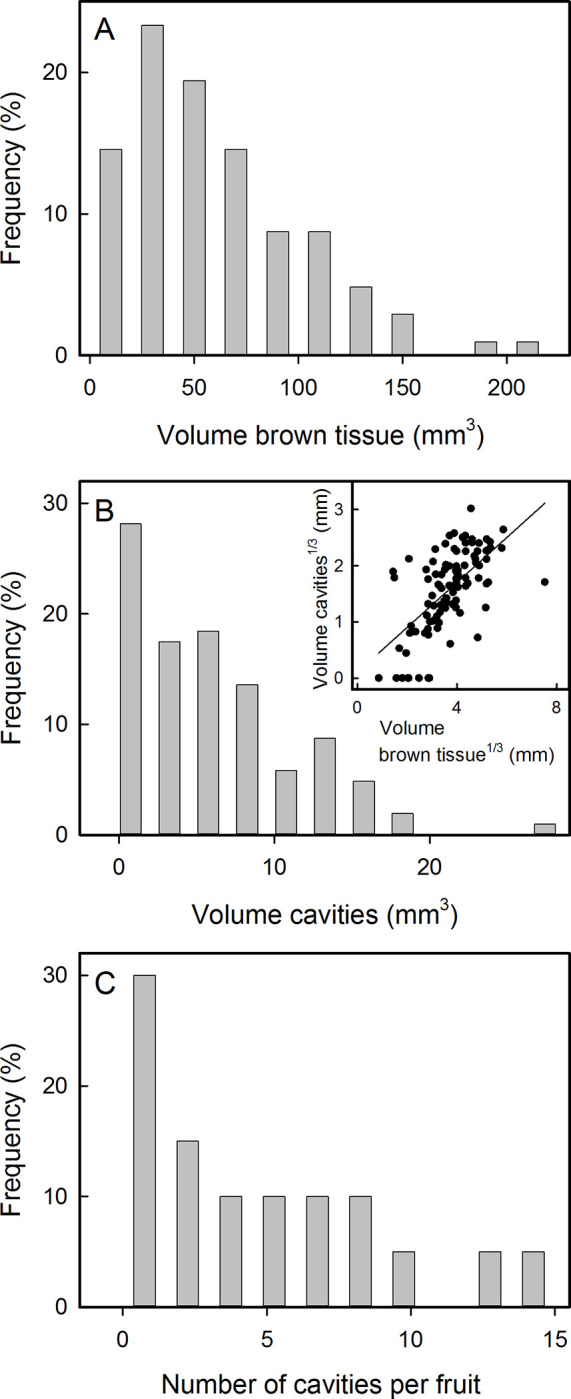
Frequency distribution of the tissue volumes with browning (A), of the volume of cavities, (B) and of the number of cavities (C) in mature symptomatic ‘Nicoter’ apples. The number of fruit inspected with tissue browning and/or cavities was 103 in (A) and (B), and 20 in (C). Inset in (B): Relationship between the volume of the brown tissue cavity and the volume of surrounding the cavity, volumes were cube root transformed (*n* = 103).

The frequency distribution of the extent of tissue browning in symptomatic ‘Nicoter’ apples revealed severe tailing towards larger tissue volumes. The most abundant volume of brown tissue was around 30 mm^3^ with few exhibiting markedly larger volumes (Fig 3A). The volume of the cavities was always smaller than the volume affected by tissue browning. Most cavities were small, but some apples had large cavities (Fig 3B). The volume of cavities and that of the surrounding brown tissue were positively and significantly correlated (r = 0.43***) (Fig 3B, inset). Most fruit with tissue browning had one or two cavities per fruit, but in a few cases severely affected fruit had up to 14 cavities per fruit (Fig 3C).

**Fig 4:**
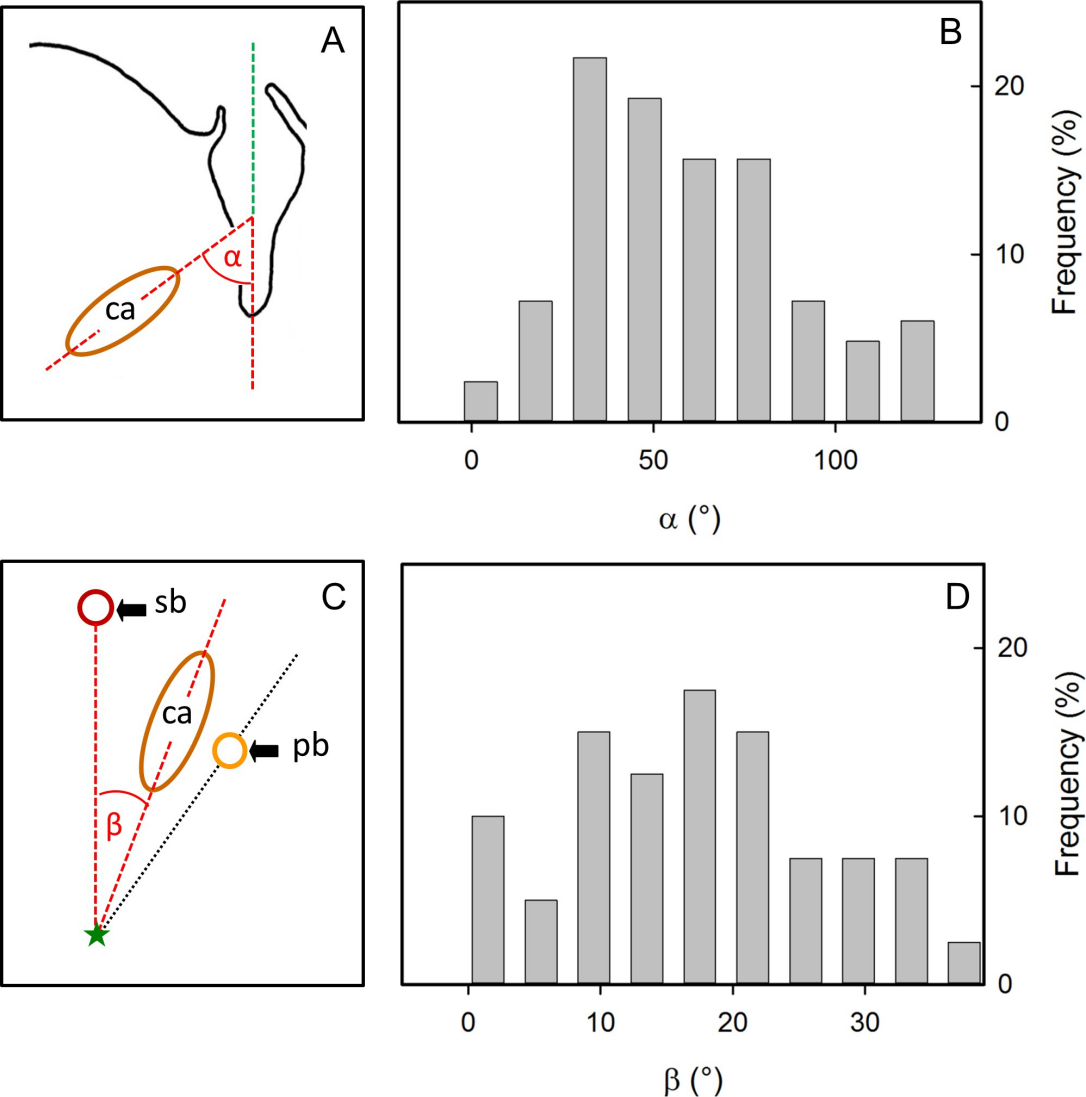
Schematic drawings (AC) and frequency distributions of the angles of orientation of cavities (BD) in mature ‘Nicoter’ apples. (AB) Orientation of cavities (ca) relative to the calyx/pedicel axis (green dashed line, asterisk); *n* = 83. (CD) Orientation of cavities (ca) in cross-section relative to the position of a bundle leading to the sepals (outer ring, see Fig 1C) or petals (inner ring, see Fig 1C); *n* = 40.

The orientation of the cavities followed a characteristic pattern. Their longitudinal axis was diagonally orientated towards the calyx-stem axis of the fruit (59.0 ±3.2°, Fig 4A and 4B). The cavities always occurred in the vicinity of the point of inflection of the vascular bundles. The radial orientation of the cavities was well-defined and corresponded to the bisecting line between the two radii that extended from the calyx/pedicel axis to the sepal and petal bundles. The bisecting line had an average angle of 17.5 ±1.5° to either one of the radii (Fig 4C and 4D).

Apple seeds are a site of auxin production and auxin plays an important role in apple fruit development [22, 23]. The number of seeds per symptomatic fruit averaged to 3.0 ± 0.3 (S1B Fig). There was no systematic relationship between the number of cavities in a sector of the calyx region and the number of seeds in the underlying locule (S1C Fig), or in the underlying plus both the neighboring locules (S1D Fig), or in the neighboring locules (S1E Fig) or in the two opposite locules (S1F Fig).

**Fig 5:**
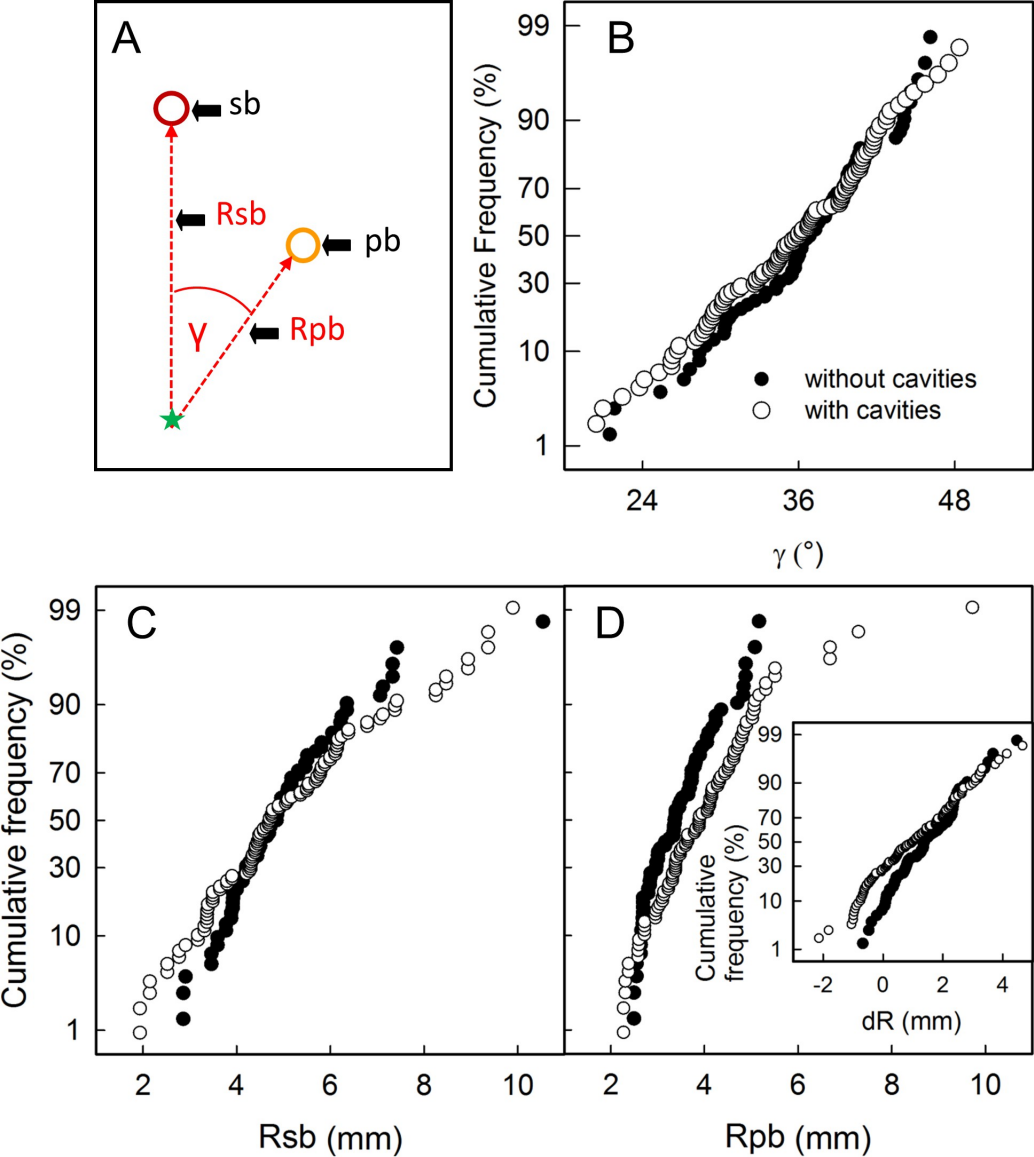
Schematic drawing (A) illustrating the position of sepal bundles (sb) and petal bundles (pb) in a cross-section in the calyx region of ‘Nicoter’ apples. Rsb, radii of sepal bundles; Rpb, radii of petal bundles; γ, angle between neighboring sepal and petal bundles; asterisk, calyx/pedicel axis. (BC) Normal probability plots of the angle between sepal bundles and petal bundles (B), of the radii of sepal bundles (C) and that of petal bundles (D) and of the difference in radii of neighboring sepal and petal bundles (dR; inset in D). Data were obtained from fruit without (*n* = 72) and with cavities (*n* = 108).

Furthermore, there were no differences in the positions of the vascular bundles supplying the sepals and petals of symptomatic vs. asymptomatic fruit (Fig 5). The angles of the radii between the sepal and petal bundles averaged 36.0 ± 0.5°, which is practically identical to the value of 360° divided by 10 bundles which represents the sum of five sepal plus five petal bundles (Fig 5B). The mean radii for the sepal bundles did not differ between symptomatic and asymptomatic fruit (5.08 ± 0.17 vs. 5.03 ± 0.17 mm, P = 0.83). However, the mean radii for the petal bundles (4.04 ± 012 vs. 3.50 ± 0.10 mm, P = 0.002) were significantly larger in symptomatic as compared to asymptomatic fruit, whereas the difference in radii between the sepal and the petal bundles (1.05 ± 0.07 vs. 1.53 ± 017 mm, P < 0.001) was smaller in symptomatic fruit (Fig 5C and 5D). Generally, the position of the bundles was more variable in symptomatic than in asymptomatic fruit.

### Immunolabelling

**Fig 6:**
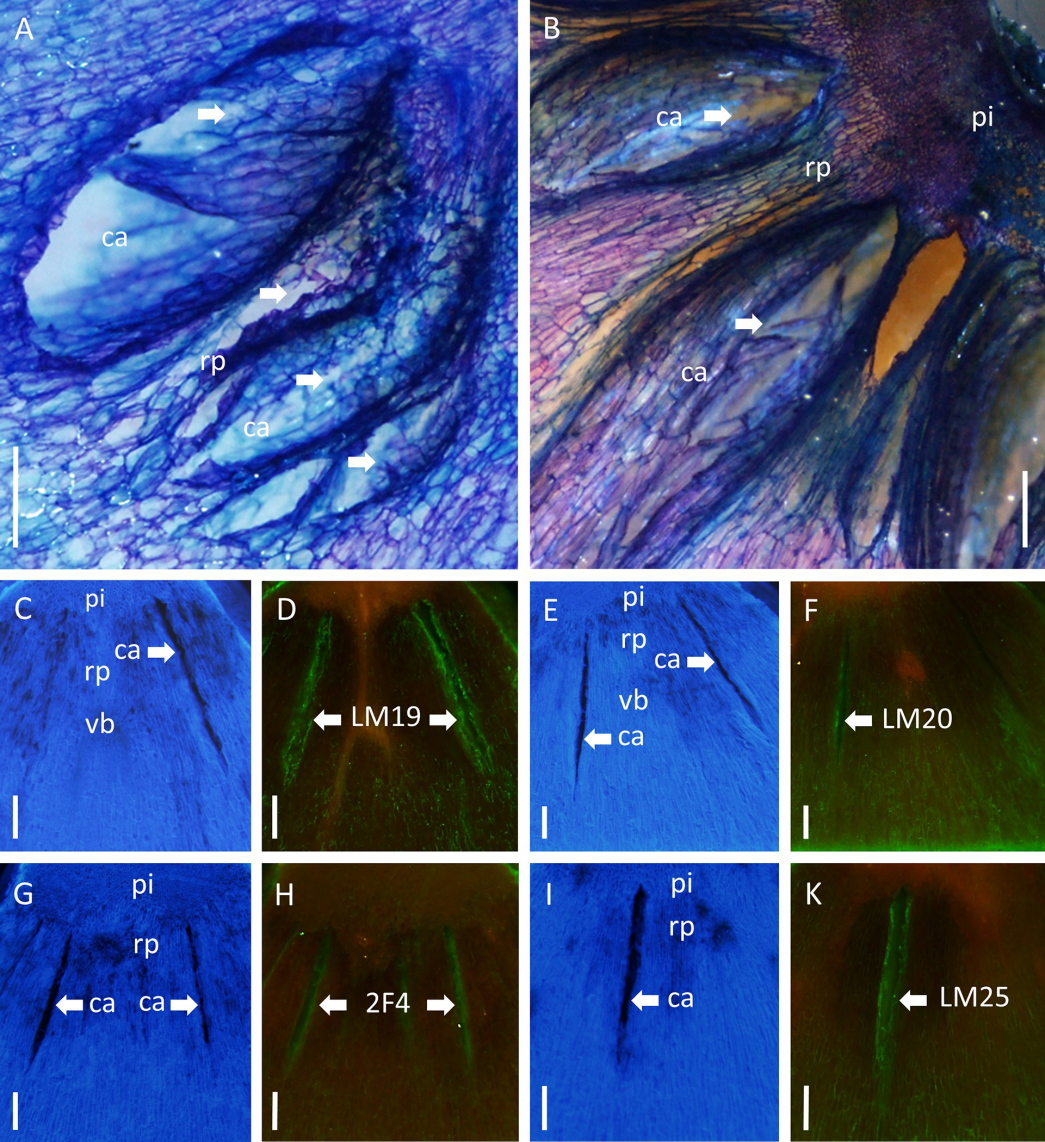
Light (AB) and fluorescence (C-K) micrographs of sections of symptomatic ‘Nicoter’ apple fruit. Longitudinal section (A) and cross-section in the calyx region (B) showing cavities (ca) that formed by rupture (arrows) of cell walls. Sections were stained with toluidine blue and subsequently viewed in incident light. pi, pith; rp, ray parenchyma.

Microscopy revealed cell wall fragments in the cavities indicating physical rupture of all cell wall layers when forming cavities (Fig 6A and 6B). The region surrounding the cavities consistently stained with calcofluor white indicating the presence of cellulose in the cell walls (Fig 6C, 6E, 6G and 6I). The rim of the cavities was strongly fluorescing when treated with LM19 that binds to demethylated homogalacturonan. The fluorescence signal obtained with LM20 (esterified homogalacturonan), 2F4 (Ca-homogalacturonan dimers) and LM25 (xyloglucan) was slightly weaker indicating a lower density of the respective epitopes on the cavity surface (Fig 6D, 6F, 6H and 6K).

Fluorescence micrographs of cross-sections (C-K): Excited with UV and blue light (for filters see material and methods) after staining with calcofluor white (CEGI) and after treatment with monoclonal antibodies (DFHK) showing cross-sections through cavities of symptomatic ‘Nicoter’ apples. The sections were stained with calcofluor white to identify cellulose (blue staining in CEGI) or reacted with antibodies against specific cell wall epitopes followed by subsequent immunolabelling (DFHK). The antibodies identified specific cell wall epitopes. The antibodies and the corresponding epitopes were: LM19 and LM20, homogalacturonans [15] (DF), 2F4 dimeric associations of Ca with homogalacturonan [16] (H) and LM25 xyloglucans [19] (K). Cavities (ca) indicated by white arrows; pi, pith; rp, ray parenchyma; vb, vascular bundle. Scale bars in (A-K) 0.5 mm. The number of fruit inspected was 25 for AB and three for C-K.

Pectic and hemicellulosic epitopes in the cell wall of ray and cortex parenchyma of the calyx region did not differ between symptomatic and asymptomatic fruit (Table 3). Fluorescence microscopy indicated the presence of homogalacturonans of different degrees of esterification (LM19, LM 20), Ca-crosslinked homogalactoronanans (2F4) and xyloglucans (LM25). Partially methyl-esterified homogalacturonans at cell junctions (LM7), xylogalacturonans (LM8), xyloarabinoxylans (LM11) or mannans (LM21) were not detected.

**Table 3:** Rating of fluorescence staining in cross-sections of ‘Nicoter’ apples, without and with cavities. Fruit were harvested when the frequency of symptomatic fruit averaged 54%, six weeks after the optimum harvest date. The tissue investigated was in the ray/cortex transition zone in a region without browning and without cavities. The antibodies against pectins were: LM7 (anti-homogalacturonan) [13], LM8 (anti-xylogalacturonan) [14], LM19 (anti-homogalacturonan) [15], LM20 (anti-homogalacturonan) [15] and 2F4 for dimeric associations of Ca with homogalacturonan [16]. Those reacting with hemicelluloses were: LM11 (anti-xylan/arabinoxylan) [17], LM21 (anti-mannan) [18] and LM25 (anti-xyloglucan) [19]. The GFP fluorescence was rated on a three-point scale: 0 no fluorescence, + some cell walls fluorescing, ++ all cell walls fluorescing; *n* = 3.

Antibody	fruit without cavities	fruit with cavities	
	rays, cortex	rays, cortex	cavities
LM7	0	0	0
LM8	0	0	0
LM19	+	++	++
LM20	++	++	+
2F4	+	++	+
LM11	0	0	0
LM21	0	0	0
LM25	++	++	++

Immunolabelling of simulated fracture surfaces produced by manually tearing specimens revealed similar composition of the cell wall on the fracture for ‘Braeburn’, ‘Gala’ and ‘Nicoter’ irrespective of harvest date (Table 4). The distribution of pectic and hemicellulosic epitopes did not differ from that described above (Table 3, Fig 6).

**Table 4:** Rating of fluorescence staining of fracture surfaces of skin strips excised from the calyx region of ‘Nicoter’, ‘Gala’ and ‘Braeburn’ apples. Fruit were harvested at the optimum harvest date and 6 weeks thereafter. The regions were: A = pith distal, B = pith proximal, C = cortex distal, D = cortex proximal; distal or proximal regions were on the level or below the calyx cavity, comprising the area above or below the inflexion point (ip) of the vascular bundle, respectively; the cortex included the ray parenchyma embedding the vascular bundles. The monoclonal antibodies (mAB) against pectins were: LM7 (anti-homogalacturonan) [13], LM8 (anti-xylogalacturonan) [14], LM19 (anti-homogalacturonan) [15], LM20 (anti-homogalacturonan) [15] and 2F4 for dimeric associations of Ca with homogalacturonan [16]. Those reacting with hemicelluloses were: LM11 (anti-xylan/arabinoxylan) [17], LM21 (anti-mannan) [18]; Marcus et al., 2010) and LM25 (anti-xyloglucan) [19]. The GFP fluorescence was rated on a three-point scale: 0 –no fluorescence, + some cell walls fluorescing, ++ all cell walls fluorescing; *n* = 3.

Harvest date	mAB	Nicoter				Gala				Braeburn			
		Region				Region				Region			
		A	B	C	D	A	B	C	D	A	B	C	D
Optimum maturity for storage	LM7	0	0	0	0	0	+	0	0	+	0	0	0
	LM8	0	0	0	0	0	0	0	0	0	0	0	0
	LM19	+	+	+	+	++	++	++	++	++	+	+	+
	LM20	++	++	++	++	++	++	++	++	+	++	++	++
	2F4	+	+	+	+	+	+	+	+	+	+	+	+
	LM11	0	0	0	0	0	0	0	0	0	0	0	0
	LM21	+	+	0	0	+	+	0	0	+	0	0	0
	LM25	++	++	++	++	++	++	++	++	++	++	++	++
Delayed by 6 weeks	LM7	0	0	0	0	0	0	0	0	++	+	+	+
	LM8	0	0	0	0	0	0	0	0	0	0	0	0
	LM19	++	++	+	+	++	++	++	++	++	++	+	+
	LM20	+	++	++	++	++	++	++	++	++	++	++	++
	2F4	+	+	+	+	++	++	++	++	+	+	+	+
	LM11	0	0	0	0	0	0	0	0	0	0	0	0
	LM21	0	0	0	0	+	0	0	0	0	0	0	0
	LM25	++	++	++	++	++	++	++	++	+	++	++	++

### Growth analysis

**Fig 7:**
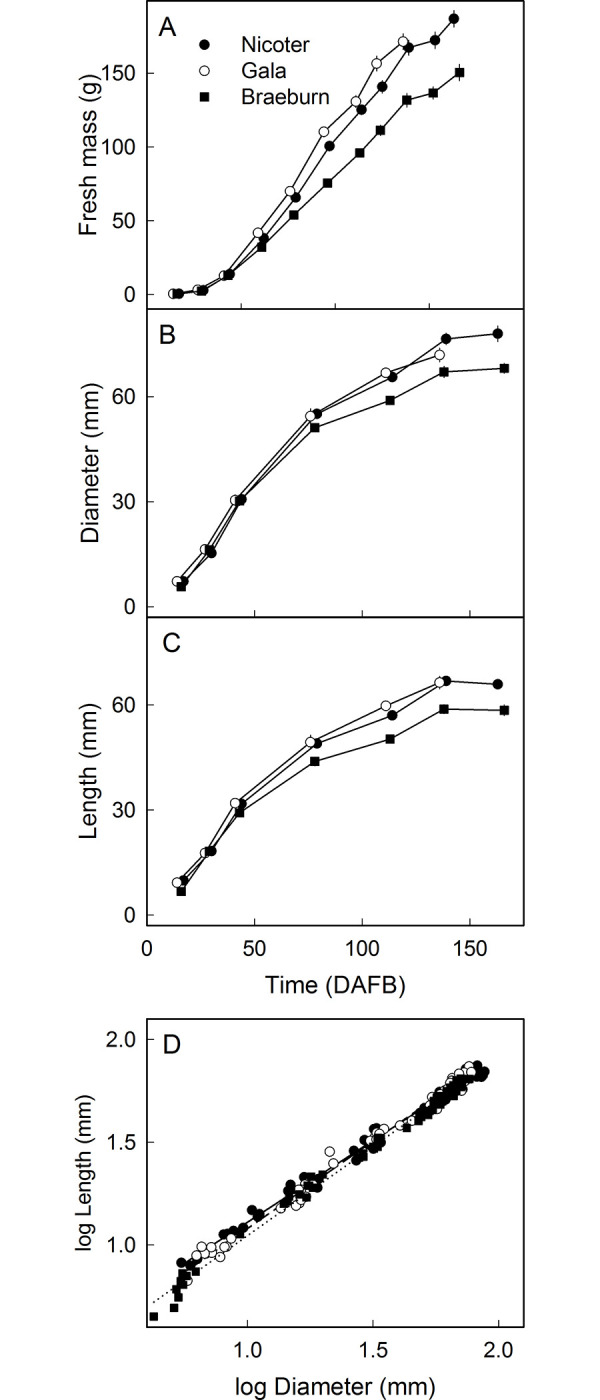
Time course of change in mass, *n* = 49–50 (A), diameter, *n* = 10 (B), length, *n* = 10 (C) of ‘Nicoter’, ‘Gala’ and ‘Braeburn’ apples. (A-C) X axis scale in days after full bloom (DAFB). (D) Plot of log length vs. log diameter, *n* = 70 (‘Nicoter’, ‘Braeburn’), *n* = 60 (‘Gala’).

The masses of ‘Nicoter’, ‘Gala’ and ‘Braeburn’ fruit increased with time in an expolinear fashion (Fig 7A). There was no difference in the growth patterns among fruit of the three cultivars but ‘Braeburn’ fruit were slightly smaller than those of ‘Gala’ and ‘Nicoter’.

The maximum diameter and maximum length of the fruit increased asymptotically. Again, ‘Braeburn’ was somewhat smaller than ‘Gala’ and ‘Nicoter’ (Fig 7B and 7C). Plotting the logarithm of fruit length against the logarithm of fruit diameter yielded linear regression lines with slopes of 0.80 ± 0.01 (‘Nicoter’, r^2^ = 0.99***), 0.85 ±y 0.01 (‘Gala’, r^2^ = 0.99***) and 0.87 ± 0.01 (‘Braeburn’; r^2^ = 0.99***). The slopes of these regressions correspond to the ratio of the growth rates of the fruit in length and diameter (Fig 7D).

**Fig 8:**
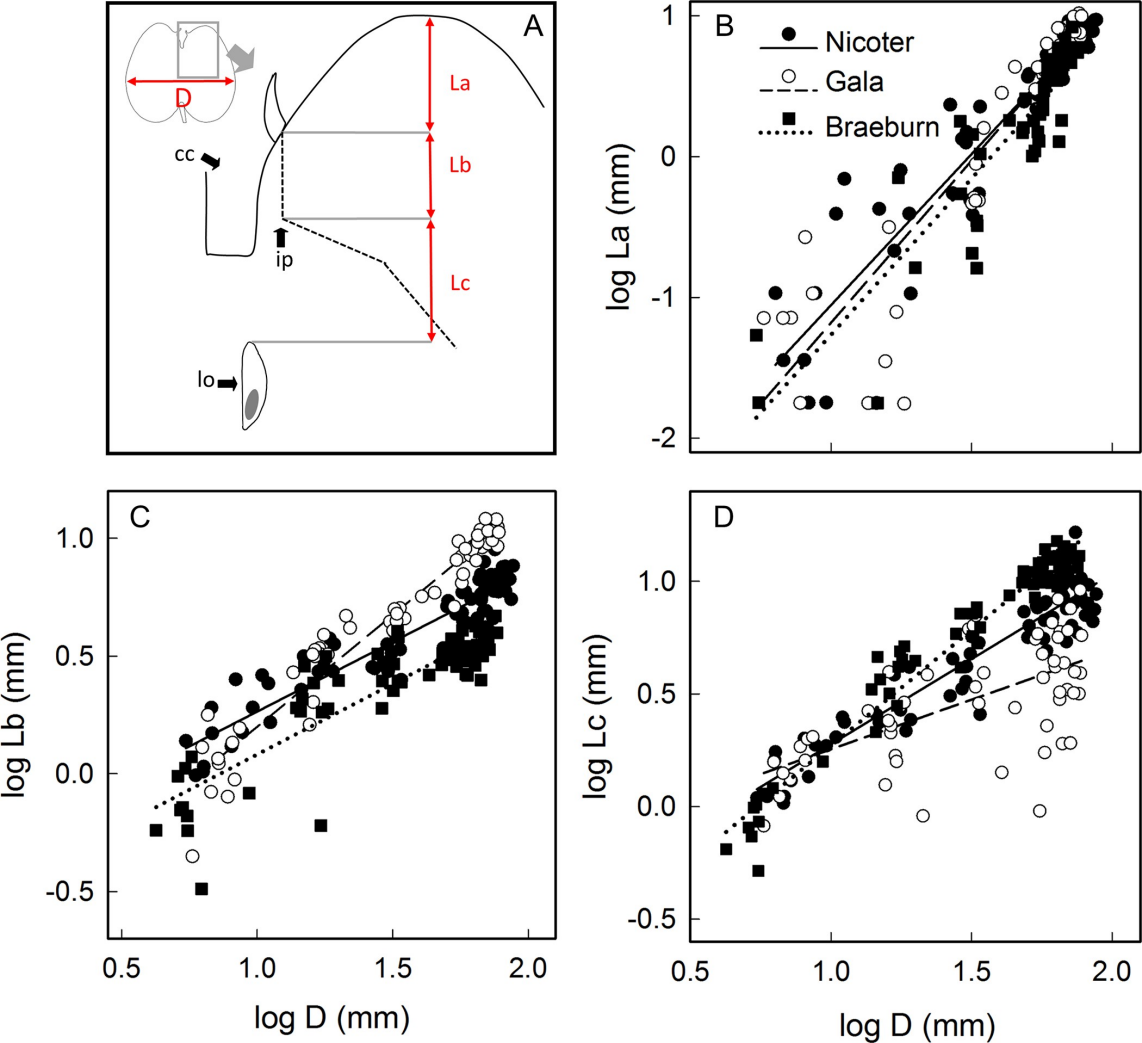
Allometric relationships between different longitudinal measures in the calyx region of ‘Nicoter’ (*n* = 70), ‘Gala’ (*n* = 60) and ‘Braeburn’ apples (*n* = 70). (A) Sketch of longitudinal section. D, maximum fruit diameter; La, distance between calyx lob and base of sepal; Lb, distance between base of sepal and point of inflection of vascular bundle (ip); Lc, distance between point of inflection of vascular bundle and distal end of locule (lo). (B-D) Allometric relationships between the logarithms of La and of D (B), between the logarithms of Lb and of D (C) and the logarithm of Lc and of D (D). For regression equations see Table 5.

Partitioning growth in length of the fruit in the calyx region into the length of the calyx lobe, the length between the base of the sepals and the point of inflection of the vascular bundle and the length between the inflection point of the vascular bundle and that of the locule revealed positive linear relationships (Fig 8A–8D). The constant differential growth ratio did not differ consistently between ‘Braeburn’, ‘Gala’ and ‘Nicoter’ (Fig 8B–8D, Table 5).

**Table 5:** Parameters of linear regression equations describing the allometric relationships in ‘Nicoter’, ‘Gala’ and ‘Braeburn’ apples between the logarithms of the distance between the calyx lobes and the base of the sepals (La) and the maximum fruit diameter, between the logarithms of the distance between the base of the sepals and the point of inflection of the vascular bundle (Lb) and the maximum fruit diameter, and between the logarithms of the distance between the point of inflection of the vascular bundles and the distal end of the locule (Lc) and the maximum fruit diameter. The data were obtained between 17 and 163 days after full bloom (DAFB) in ‘Nicoter’ (*n* = 70), 14 and 136 DAFB in ‘Gala’ (*n* = 60), and 16 and 166 DAFB in ‘Braeburn’ (*n* = 70). For details see Fig 8.

Cultivar	Parameter	Regression parameters		Coefficient of determination
		Intercept	Slope	(r^2^)
Nicoter	La	-3.20±0.18	2.15±0.11	0.87***
	Lb	-0.33±0.04	0.59±0.03	0.88***
	Lc	-0.48±0.05	0.76±0.03	0.89***
Gala	La	-3.48±0.24	2.30±0.15	0.84***
	Lb	-0.75±0.05	0.95±0.03	0.93***
	Lc	-0.19±0.12	0.44±0.08	0.37***
Braeburn	La	-3.48±0.27	2.22±0.16	0.80***
	Lb	-0.52±0.06	0.60±0.04	0.77***
	Lc	-0.76±0.04	1.03±0.03	0.96***

**Fig 9:**
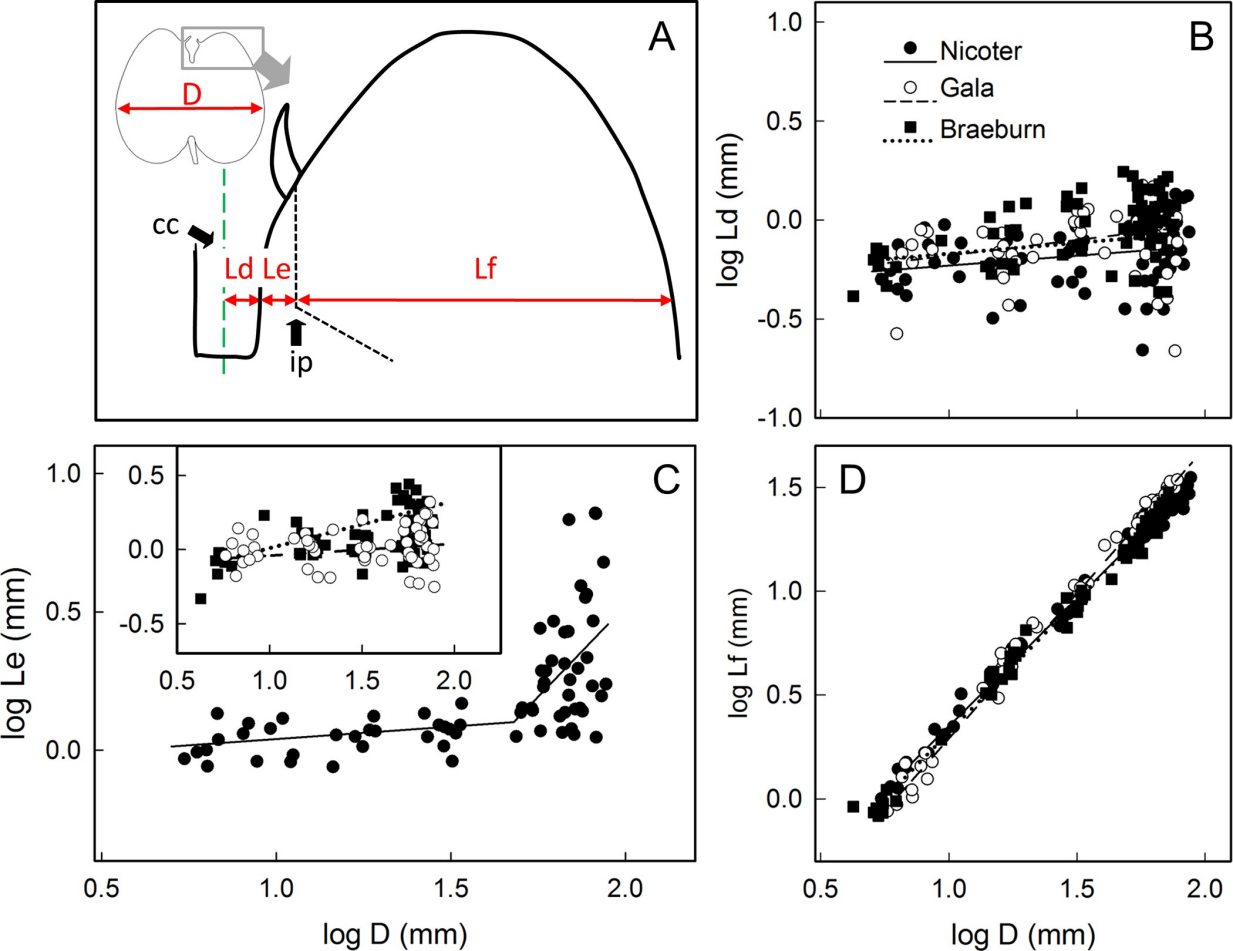
Allometric relationships between different radial measures in the calyx region of ‘Nicoter’ (*n* = 70), ‘Gala’ (*n* = 60) and ‘Braeburn’ apples (*n* = 70). (A) Sketch of longitudinal section. D, maximum fruit diameter; Ld, distance between calyx-pedicel axis and surface of calyx cavity; Le, distance between surface of calyx cavity and point of inflection of vascular bundle (ip); Lf, distance between point of inflection of vascular bundles and fruit surface. (B-D) Allometric relationships between the logarithms of Ld and of D (B), between the logarithms of Le and of D (C) and the logarithms of Lf and of D (D). For regression equations see Table 6.

The same analysis for fruit diameter in the calyx region (Fig 9, Table 6) revealed little increase in diameter of the calyx cavity in ‘Nicoter’, ‘Braeburn’ or ‘Gala’ (Fig 9A and 9B). The diameter change of the calyx cavity was slow and occurred at comparable rates in all three cultivars (Fig 9B). However, in ‘Braeburn’ and ‘Gala’, the portion of the diameter between the calyx surface and the point of inflection of the vascular bundles increased consistently as fruit diameter increased (Fig 9C inset). In contrast, in ‘Nicoter’ there was almost no growth in this region until the value of the log of fruit diameter reached about 1.6 (equivalent to about 43 mm diameter at 60–62 DAFB). Thereafter, fruit growth in ‘Nicoter’ was rapid and highly variable (Fig 9C). There were no differences between the three cultivars in the growth rates of the region between the vascular bundles and the peel—all grew at similar rates. The rate of growth in the calyx region was larger than that in the equatorial plane (Fig 9D).

**Table 6:** Parameters of regression equations describing allometric relationships in ‘Nicoter’, ‘Gala’ and ‘Braeburn’ apples between the logarithms of the distance between the calyx-stem axis and the surface of the calyx cavity (Ld) and the maximum fruit diameter, between the logarithms of the distance between the surface of the calyx cavity and the point of inflection of the vascular bundles (Le) and the maximum fruit diameter, and the logarithms of the distance between the point of inflection of the vascular bundle and the fruit surface (Lf) and the maximum fruit diameter. Linear regression models were used throughout. The only exception was for Le in ‘Nicoter’, where a segmented linear regression model was used. The data were obtained between 17 and 163 days after full bloom (DAFB) in ‘Nicoter’ (*n* = 70), 14 and 136 DAFB in ‘Gala’ (*n* = 60) and 16 and 166 DAFB in ‘Braeburn’ (*n* = 70). For details see Fig 9.

Cultivar	Variable	Time period	Regression parameters		Coefficient of determination	Intercept of segmented regression line
		(DAFB)	Intercept	Slope	(r^2^)	
Nicoter	Ld	17–163	-0.33±0.08	0.10±0.05	0.06n.s.	
	Le	17–46	-0.05±0.13	0.09±0.11	0.54***	1.66
		47–163	-2.06±0.69	1.29±0.38		
	Lf	17–163	-0.88±0.02	1.23±0.01	0.99***	
Gala	Ld	14–136	-0.28±0.10	0.11±0.06	0.06n.s.	
	Le	14–136	-0.12±0.07	0.08±0.05	0.06n.s.	
	Lf	14–136	-1.11±0.02	1.40±0.02	0.99***	
Braeburn	Ld	16–166	-0.38±0.07	0.22±0.04	0.27***	
	Le	16–166	-0.24±0.07	0.22±0.04	0.28***	
	Lf	16–166	-0.95±0.02	1.27±0.01	0.99***	

### Tensile tests

Representative stress/strain diagrams of the parenchyma tissue excised from the cortex of ‘Braeburn’, ‘Gala’ and ‘Nicoter’ fruit revealed a similar modulus of elasticity for all three cultivars (range 16.8 to 24.3 N). Stress at fracture and strain at fracture were highest for ‘Braeburn’ and ‘Nicoter’, and lowest for ‘Gala’ (S3 Fig; S1 Table).

## Discussion

The results indicate: (1) that vascular browning of ‘Nicoter’ apple is accompanied by the formation of cavities and (2) that differential growth rates of pith, ray parenchyma and cortex of ‘Nicoter’ apple result in tissue tensions and tissue failure in the ray parenchyma and cortex that are absent in both of the parent cultivars.

### Vascular browning, tissue browning and cavity formation–definition and characteristics

In horticultural practice, the disorder of ‘Nicoter’ is referred to as vascular browning [2]. It describes the browning of the internal tissues and the formation of cavities in the calyx end and/or in the stem end of that occur late in fruit development (Fig 1). There are no comparable symptoms in either of the parent cultivars, ‘Gala’ and ‘Braeburn’. Browning of tissue typically results from a loss of compartmentation due to cell rupture and cell death [14]. When ruptured, the polyphenoloxidases from the cytoplasm come into contact with monophenols and diphenols from the vacuole which are soon oxidized to quinones. These, then polymerize to form brown polyphenols [24]. The intercellular cavities become gas filled, rather than liquid filled. Cavity number and size both increase as the severity of vascular browning symptoms increases (Fig 3). Tissue browning always occurs at the interface between a cavity and the healthy tissue around it. This explains why cavity formation is always associated with tissue browning.

In our study we focused on vascular browning in the calyx end, since this occurs more frequently than in the stem end (Table 1). However, there is no evidence that vascular browning at the stem end is in any way different from that in the calyx end, with identical symptoms. Tissue browning and cavity formation extend from the ray parenchyma between the vascular bundles into the cortex. The pith and the vascular bundles are generally not affected by browning (Fig 1). We found no evidence that the vasculature was involved in this disorder, so the naming of the disorder ‘vascular browning’ is misleading. The cavities occurred in a region of the fruit where cells were subject to rapid rates of extension. This typically occurs near the inflection point of the vascular system in all three cultivars—‘Nicoter’, ‘Braeburn’ and ‘Gala’. Here the cells are highly anisodiametric, with the direction of cell extension differing markedly over quite small distances, indicating marked changes in the direction of tissue strain over quite short distances (Fig 2). In materials science, the analysis of fracture patterns often provides useful information on the mechanism of fracture. Applying this approach to symptomatic ‘Nicoter’ apples, the shapes and orientations of the cavities allow us to infer the following: (1) Cavity formation probably begins at the intersection of the three orthogonal axes of the cavity (the longitudinal and the two latitudinal axes). The spindle shape then results from ongoing lateral growth strains, perpendicular to the longitudinal axis of the cavity. As a result, the stresses concentrate at the polar tips of the cavity, causing the cavity to extend longitudinally. (2) Based on the position of the cavities in the fruit, the ray parenchyma and the cortex between the sepal and petal bundles at, or slightly below, the calyx cavity are the most likely origins of cavity formation. (3) The downward orientation of the cavities results from longitudinal extension of the calyx end of the fruit in the direction of the central axis, relative to the surrounding tissue. This interpretation is consistent with the marked longitudinal elongation of cells in the pith and the lack thereof in the cortex. Instead, the cells of the ray parenchyma and cortex elongate in a latitudinal direction.

### The mechanistic basis of vascular browning of ‘Nicoter’

**Fig 10:**
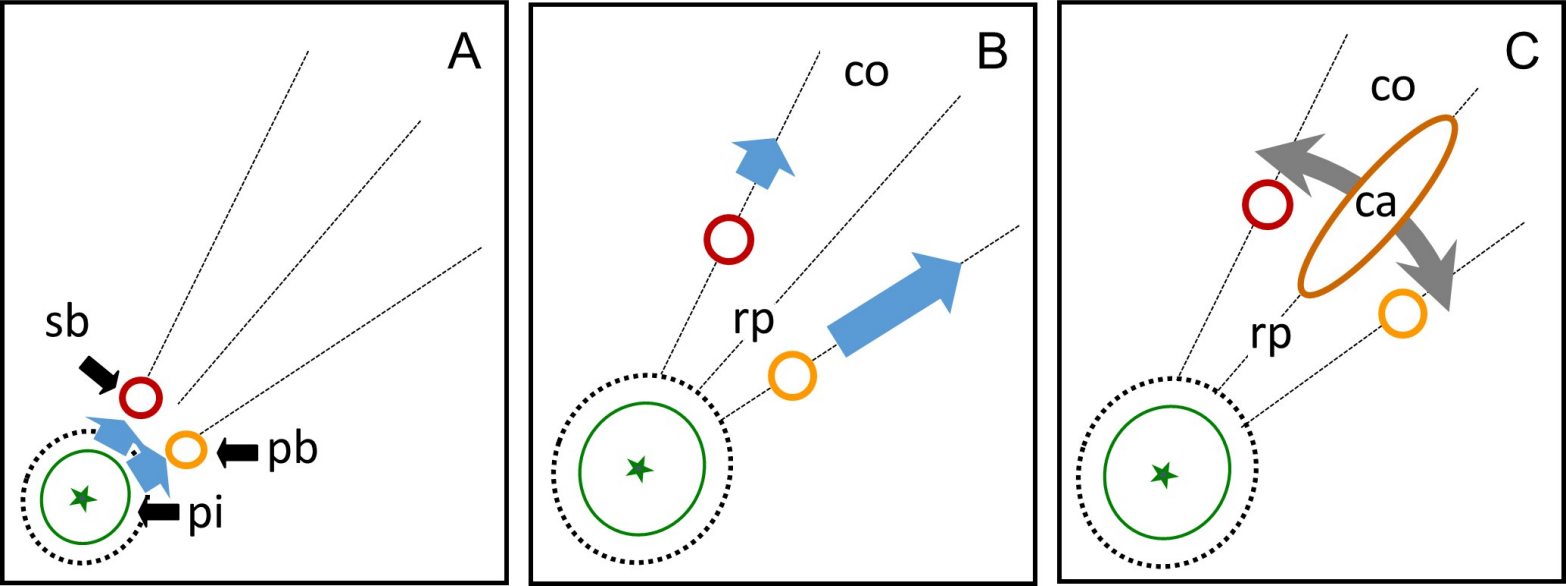
Sketch of cross-section of ‘Nicoter’ apples in the calyx region illustrating development of the vascular browning disorder. (A) Growth of the pith (pi) displaces the sepal bundles (sb) and in particular the petal bundles (pb). (B) The outward movement of the vascular bundles is particularly large for the petal bundles and the ray parenchyma (rp). (C) The diverging bundles subject the ray parenchyma between the vascular bundles to tangential growth stress normal to the calyx/stem axis. The stress-induced strain results in rupture of the cortex (co) as indexed by tissue browning and cavity formation (ca). Arrows in (A-C) indicate directions of stress and strain.

Our results suggest vascular browning results from a growth anomaly, specific to ‘Nicoter’ apples, that does not occur in either of its parents—‘Braeburn’ or ‘Gala’. From 60 to 62 DAFB onwards, the growth of the pith and ray parenchyma in the calyx region push the vascular bundles, particularly the petal bundles and, less so, the sepal bundles radially outwards (Fig 10A). This movement is not affected by auxins produced by the seeds (S1 Fig) or by exogenous Promalin (Table 1). The outward movement is highly variable as indexed by the normal probability plots of the radius of the sepal bundles in symptomatic vs. asymptomatic fruit (Fig 5C) and the high variability of the allometric relationships depicted in Fig 10C. The radial outward movement of the vascular bundles increases the distance (latitudinally) between the bundles thereby subjecting the ray parenchyma and cortex between the bundles to latitudinal tangential strain (Fig 10B). The strain causes the failure of the cell walls and the formation of the cavities (Fig 10C).

Several features indicate that physical rupture is the mode of cavity formation. First, the tissue browning associated with cavity formation indicates the rupture of cell walls, an associated loss of compartmentation and the classical polyphenoloxidase wound reaction. Second, the release of malic acid into the apoplast, associated with cell wall rupture, will extract Ca from the cell wall. This results in a weakening of the adjacent cell walls, and thus the progressive (a ‘chain reaction’) rupture of the tissue resulting in crack extension–similar to the cracking observed in sweet cherry fruit [12, 25, 26]. Third, light microscopy revealed cell wall fragments, indicative of cell wall rupture. Fourth, there was no difference in the immunolabelling of fracture surfaces of cortex tissue when artificial fracture surfaces were created by cutting or by tearing. If cavities formed by separation of adjacent cells (fracture mode along cell walls), differential immunolabelling of surfaces generated by cutting vs. tearing would have occurred. Immunolabeling evidence for this was not found. Nor was there evidence of any differences in cell wall constituents between symptomatic and asymptomatic ‘Nicoter’ or between ‘Nicoter’ and either of its parents. Moreover, the mechanical properties of the cortex tissue of ‘Nicoter’ were similar to those of ‘Braeburn’. The cortices of these cultivars were both stiffer and stronger than that of ‘Gala’. Based on the tensile strength measured in the outer cortex tissue of ‘Nicoter’ apples, the growth stress generated and required to fracture cellulosic cell walls of our specimens must have exceeded 0.26 N mm^-2^ (equiv. to a fracture force of 4 N given the geometry of our specimen). Unfortunately, the small size of the tissue volume at the point of inflection of the vascular bundles (where cavity formation begins) did not allow excision of specimens for mechanical testing. Hence, test specimens had to be prepared from the outer cortical region.

### Vascular browning of ‘Nicoter’ is unlike other browning disorders of apple

The vascular-browning disorder of ‘Nicoter’ differs in symptoms and mechanism from other internal flesh browning disorders in apple (for a recent review see [27]). In vascular browning in ‘Nicoter’ the symptoms are limited to the tissues immediately adjacent to the vascular bundles in the calyx and the stem-end region of the fruit. The core region seems to be unaffected. Our results show that growth strain is likely the primary trigger for vascular browning. In contrast, internal flesh-browning disorders affects the flesh and the core of apples. They are typically caused by chilling injury, or internal CO_2_ injury, or senescent breakdown, but growth strain has not been identified as a factor. Furthermore, internal flesh-browning disorders typically affect a small range of apple cultivars, whereas the vascular browning disorder described here is unique to ‘Nicoter’. The only characteristic common between internal flesh-browning disorders and vascular browning in ‘Nicoter’ is the browning reaction itself that results from a membrane disruption followed by enzymatic oxidation of phenolic compounds by polyphenol oxidases (PPOs) [24, 28].

## Conclusion

Growth stress is the driving force for cavity formation in ‘Nicoter’. Rapid radial growth of the pith between the calyx surface and the vascular bundles fairly late in the growing season is causal in vascular browning. As a result, the cortex is pushed outwards. The increase in perimeter subjects the cortex to excessive latitudinal tangential strain which results in the formation of cavities. This sequence of events explains the typical radial orientation of the cavities. As there is no such growth in the pith of ‘Nicoters’ two parent cultivars—‘Gala’ and ‘Braeburn’—these remain asymptomatic. This conclusion is consistent with the increased incidence of symptomatic fruit when harvest is delayed. Vascular browning is thus a specific characteristic of ‘Nicoter’ apples. A timely harvest is probably the only practicable countermeasure for decreasing the incidence of this disorder.

## Supporting information

S1 Table Modulus of elasticity, force at fracture, and strain at fracture of cortex tissue of ‘Nicoter’, ‘Gala’ and ‘Braeburn’ apples. Mechanical properties were established in uniaxial tensile tests. Data are means ± SE.
(DOCX)

S1 Fig Longitudinal cross-section of ‘Nicoter’ apple without (A) and with symptoms of vascular browning in the calyx end (see arrows) (B). Scale bar 1 cm.
(TIF)

S2 Fig Role of seeds in vascular browning of ‘Nicoter’ apples. (A) Sketch of cross-section illustrating the position of locules (lo). (B) Frequency of symptomatic apples as affected by the number of seeds per fruit, *n* = 43. (C) Relationship between the number of seeds in the locule next to a cavity (locule 1) and the number of cavities. (D) Same as (C), but number of seeds in the three locules next to the cavities (locules 1+2+3) and the number of cavities. (E) Same as (C), but number of seeds in two neighboring locules (locules 2+3) and the number of cavities. (F) Same as (C) but number of seeds in the two locules on the opposite side of the cavities (locules 4+5). (C-F) *n* = 35.
(TIF)

S3 Fig Representative stress/strain diagrams for specimens of the outer cortex excised from mature ‘Nicoter‘, ‘Gala‘ and ‘Braeburn‘ apples. The stress strain diagrams were obtained in uniaxial tensile tests. The slope of the stress strain diagrams corresponds to the modulus of elasticity of the specimen. The moduli of elasticity were 16.8 ± 0.5 N for ‘Nicoter’, 24.3 ± 1.7 N for ‘Gala’, and 19.4 ± 0.5 N for ‘Braeburn’.
(TIF)

S1 File This is the excel file containing the data in Figs 3–9, S2 and S3 Figs, Tables 1–6 and S1 Table. (XLSX)
