# Supplementary material for: Growth strains cause vascular browning and cavities in ´Nicoter´ apples
Source: PLoS One. 2023 Jul 20;18(7):e0289013. doi: 10.1371/journal.pone.0289013 (PMC10359005; doi:10.1371/journal.pone.0289013)
Supplement: S1 Table — Mechanical properties were established in uniaxial tensile tests. Data are means ± SE. (DOCX) [file pone.0289013.s001.docx]

| Table S1. Modulus of elasticity, force at fracture, and strain at fracture of cortex tissue of ‘Nicoter’, ‘Gala’ and ‘Braeburn’ apples. Mechanical properties were established in uniaxial tensile tests. Data are means ± SE. | | | |
| --- | --- | --- | --- |
| Cultivar | Modulus of elasticity (N) | Force at fracture (N) | Strain at fracture (mm^2^ mm^-2^) |
| Nicoter | 16.8 ± 0.5 | 3.5 ± 0.2 | 0.26 ± 0.01 |
| Gala | 24.3 ± 1.7 | 1.2 ± 0.2 | 0.08 ± 0.01 |
| Braeburn | 19.4 ± 0.5 | 4.7 ± 0.2 | 0.30 ± 0.01 |
|  |  |  |  |
