# Supplementary figures and images for: Growth strains cause vascular browning and cavities in ´Nicoter´ apples
Source: PLoS One. 2023 Jul 20;18(7):e0289013. doi: 10.1371/journal.pone.0289013 (PMC10359005; doi:10.1371/journal.pone.0289013)

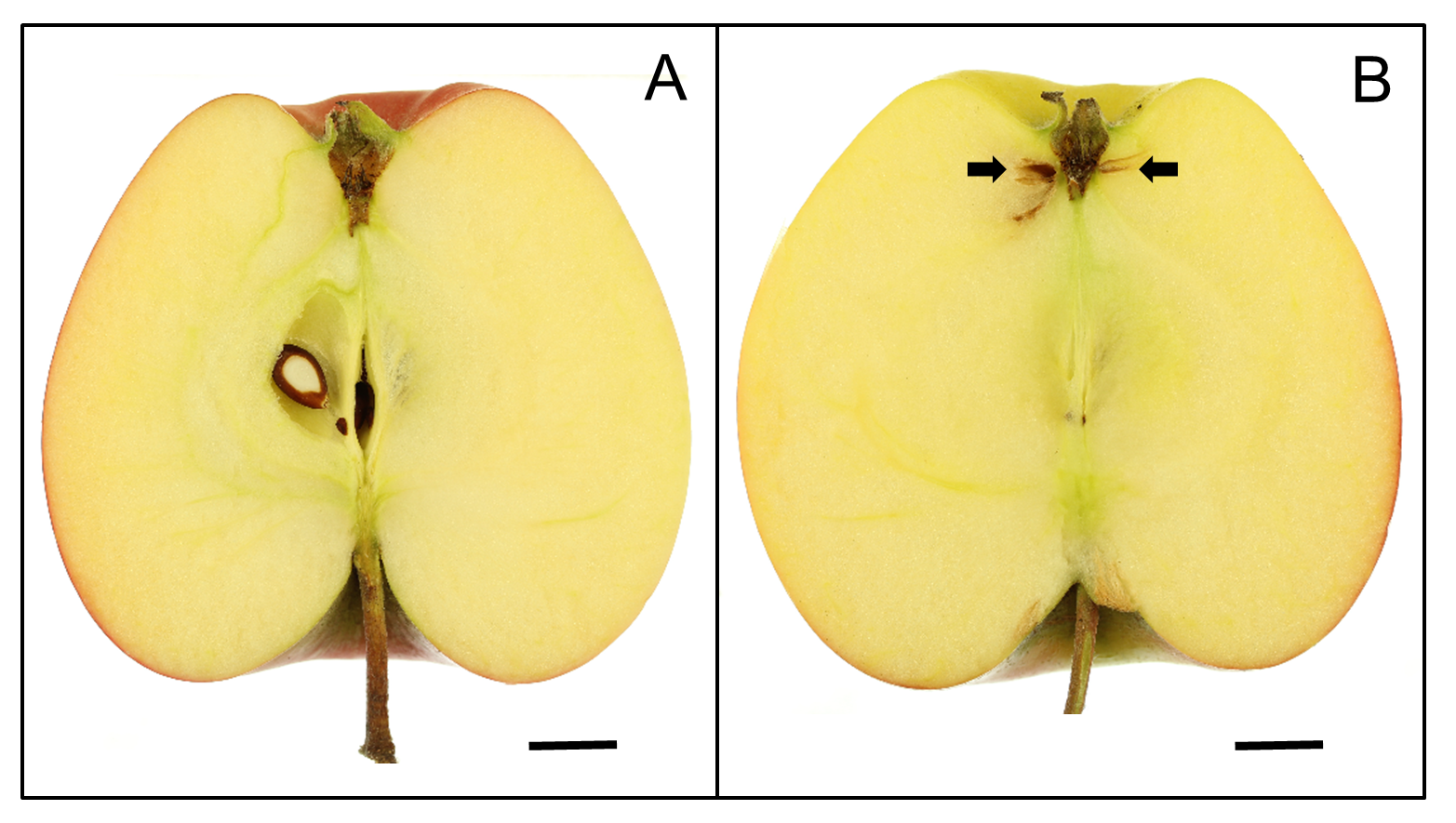

Supplement: S1 Fig — Scale bar 1 cm. (TIF) [file pone.0289013.s002.tif]

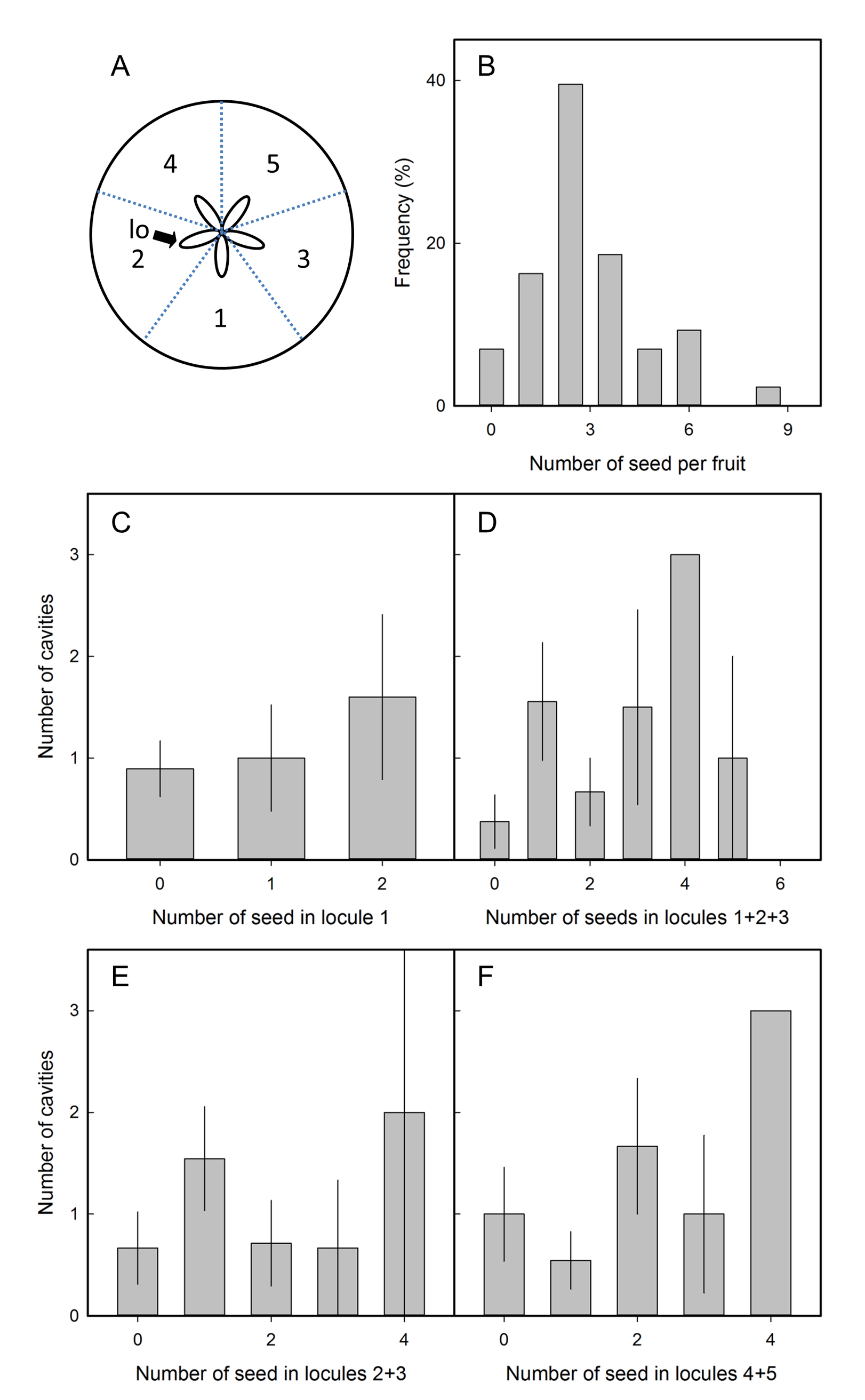

Supplement: S2 Fig — (A) Sketch of cross-section illustrating the position of locules (lo). (B) Frequency of symptomatic apples as affected by the number of seeds per fruit, n = 43. (C) Relationship between the number of seeds in the locule next to a cavity (locule 1) and the number of cavities. (D) Same as (C), but number of seeds in the three locules next to the cavities (locules 1+2+3) and the number of cavities. (E) Same as (C), but number of seeds in two neighboring locules (locules 2+3) and the number of cavities. (F) Same as (C) but number of seeds in the two locules on the opposite side of the cavities (locules 4+5). (C-F) n = 35. (TIF) [file pone.0289013.s003.tif]

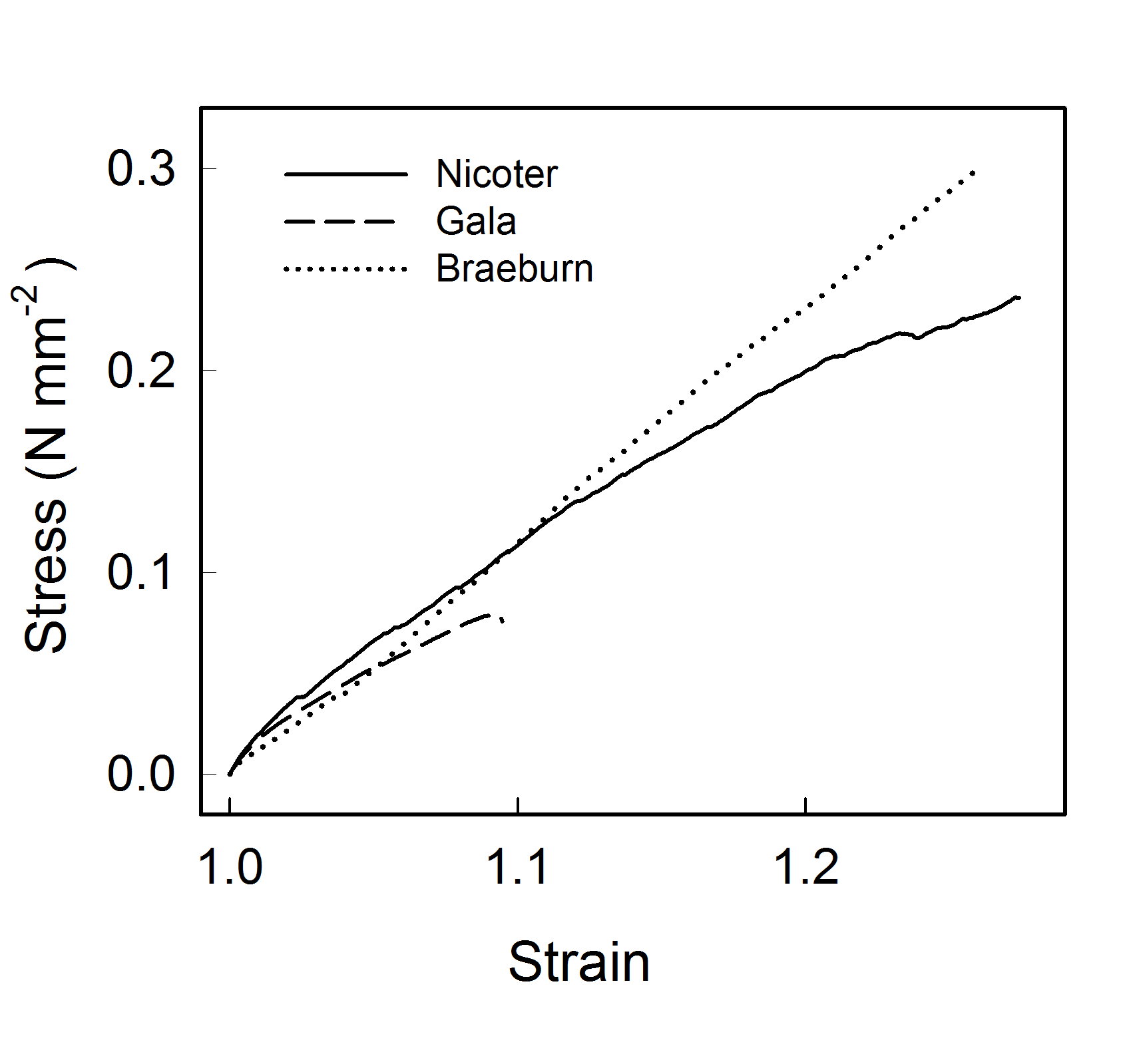

Supplement: S3 Fig — The stress strain diagrams were obtained in uniaxial tensile tests. The slope of the stress strain diagrams corresponds to the modulus of elasticity of the specimen. The moduli of elasticity were 16.8 ± 0.5 N for ‘Nicoter’, 24.3 ± 1.7 N for ‘Gala’, and 19.4 ± 0.5 N for ‘Braeburn’. (TIF) [file pone.0289013.s004.tif]
